# Reply to Argüelles and Argüelles-Prieto, “Are the Editors Responsible for Our Obsession with the Impact Factor?”

**DOI:** 10.1128/mBio.02079-17

**Published:** 2017-12-19

**Authors:** Arturo Casadevall, Ferric C. Fang

**Affiliations:** a Johns Hopkins Bloomberg School of Public Health, Baltimore, Maryland, USA; b Department of Microbiology, University of Washington, Seattle, Washington, USA

**Keywords:** editors, impact factor, publishing

## REPLY

Argüelles and Argüelles-Prieto ask an important question: are editors responsible for journal impact factor (IF) mania? We previously suggested that an obsession with publishing scientific work in a journal with the highest possible IF is primarily driven by scientists themselves, creating a tragedy of the commons regarding the allocation of scientific prestige (1). As publishing in a high-IF journal disproportionately benefits those who succeed, all scientists are compelled to play the game. However, journals can create an artificial scarcity by limiting the number of articles published, thereby ensuring that most scientists will fail. In this economic system, the publishing venue often carries more weight than the actual scientific merit of a paper, at least in the short run.

As gatekeepers of the publication process, journal editors are unavoidably complicit in maintaining the IF economy. However, just because editors must work within this reward system does not make them responsible for IF mania. The primary responsibility of journal editors is to select high-quality work for their journals and to maintain the journals’ reputation. The preoccupation with journal IF, also referred to as “impactitis” (2), is generated not by editors but rather by the scientists comprising the review committees responsible for hiring, promotion, or funding decisions, who judge scientific papers by the prestige of the journal rather than the actual quality of the work. If such committees were to focus on scientific rigor and integrity instead of journal prestige, the importance of the journal IF would disappear overnight.

Furthermore, we disagree with the statement that the “scientific community has universally accepted that the so-called ‘top journals’ only publish the most relevant papers, which are at the frontier of new knowledge.” The notion that quality can be measured by the publication venue has been emphatically rejected by the San Francisco Declaration of Research Assessment (3). In fact, we have found a correlation between the journal IF and the probability of retraction (4) and that most retractions are due to misconduct (5). Impact is not equivalent to scientific importance (6). Moreover, the distribution of the citation impact of individual papers published by high-impact journals is quite broad (7), and in many cases, the most impactful papers only achieve widespread recognition after the 2-year citation window reflected in the journal IF (8). Hence, it is difficult to argue that the papers published by journals with high IFs necessarily represent the best work.

Finally, we dispute the notion that editors should not be allowed to submit papers to the journals that they serve. Argüelles has made this argument before (9), and we continue to disagree with this suggestion (10). Editors are selected on the basis of their experience and qualifications, typically including a sustained record of publishing high-quality papers in the journals where they will serve as editors. It would be perverse to forbid them to submit future work to the journal, which would be detrimental for both the journal and the editor and make the recruitment of good editors far more challenging. To mitigate a conflict of interest, the American Society for Microbiology journal policies mandate an absolute firewall for papers submitted by editors, who are treated like any other author and blinded from the review process. To the contrary, we suggest that publishing in the journals they edit makes editors more invested in their journals and their quality.
